# *Momordica charantia* Exosome-Like Nanoparticles Exert Neuroprotective Effects Against Ischemic Brain Injury *via* Inhibiting Matrix Metalloproteinase 9 and Activating the AKT/GSK3β Signaling Pathway

**DOI:** 10.3389/fphar.2022.908830

**Published:** 2022-06-24

**Authors:** Heng Cai, Lin-Yan Huang, Rui Hong, Jin-Xiu Song, Xin-Jian Guo, Wei Zhou, Zhao-Li Hu, Wan Wang, Yan-Ling Wang, Jian-Gang Shen, Su-Hua Qi

**Affiliations:** ^1^ Pharmacology College, Xuzhou Medical University, Xuzhou, China; ^2^ Medical and Technology School, Xuzhou Medical University, And Xuzhou Key Laboratory of Laboratory Diagnostics, Xuzhou, China; ^3^ Research Center for Biochemistry and Molecular Biology and Jiangsu Key Laboratory of Brain Disease Bioinformation, Xuzhou Medical University, Xuzhou, China; ^4^ School of Chinese Medicine, The University of Hong Kong, Hong Kong, China

**Keywords:** exosome-like nanoparticles, *Momordica charantia*, blood–brain barrier, matrix metalloproteinase 9, ischemic/reperfusion injury

## Abstract

Plant exosome-like nanoparticles (ELNs) have shown great potential in treating tumor and inflammatory diseases, but the neuroprotective effect of plant ELNs remains unknown. In the present study, we isolated and characterized novel ELNs from *Momordica charantia* (MC) and investigated their neuroprotective effects against cerebral ischemia-reperfusion injury. In the present study, MC-ELNs were isolated by ultracentrifugation and characterized. Male Sprague–Dawley rats were subjected to middle cerebral artery occlusion (MCAO) and MC-ELN injection intravenously. The integrity of the blood–brain barrier (BBB) was examined by Evans blue staining and with the expression of matrix metalloproteinase 9 (MMP-9), claudin-5, and ZO-1. Neuronal apoptosis was evaluated by TUNEL and the expression of apoptotic proteins including Bcl2, Bax, and cleaved caspase 3. The major discoveries include: 1) Dil-labeled MC-ELNs were identified in the infarct area; 2) MC-ELN treatment significantly ameliorated BBB disruption, decreased infarct sizes, and reduced neurological deficit scores; 3) MC-ELN treatment obviously downregulated the expression of MMP-9 and upregulated the expression of ZO-1 and claudin-5. Small RNA-sequencing revealed that MC-ELN-derived miRNA5266 reduced MMP-9 expression. Furthermore, MC-ELN treatment significantly upregulated the AKT/GSK3β signaling pathway and attenuated neuronal apoptosis in HT22 cells. Taken together, these findings indicate that MC-ELNs attenuate ischemia-reperfusion–induced damage to the BBB and inhibit neuronal apoptosis probably *via* the upregulation of the AKT/GSK3β signaling pathway.


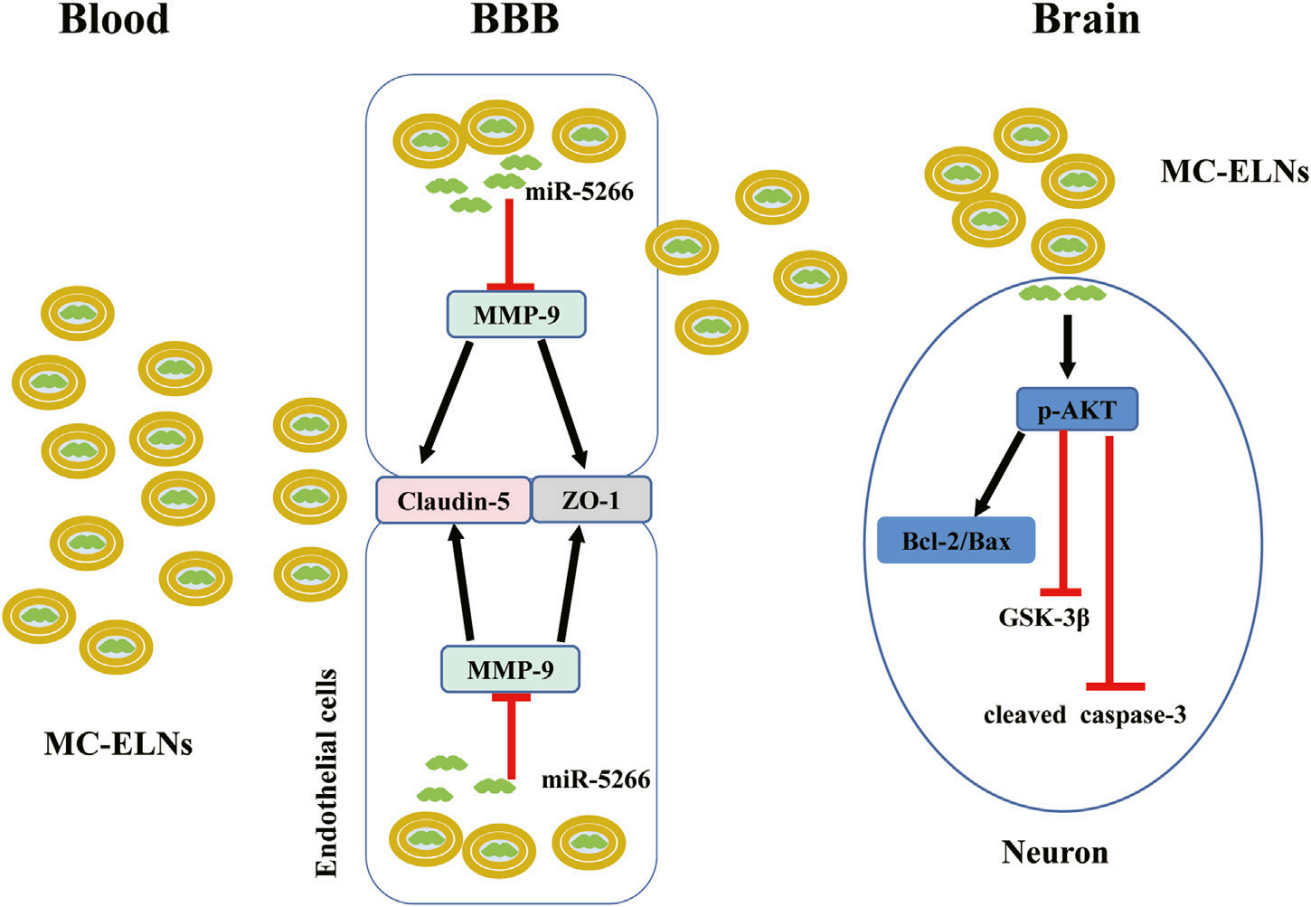


## Introduction

Stroke is the second leading cause of death and third leading cause of disability in adults worldwide (Katan and Luft, 2018). About 85% of stroke victims are ischemic with reduced blood flow, generally resulting from arterial occlusion (Musuka et al., 2015). Recombinant human tissue plasminogen activator (t-PA) remains the only Food and Drug Administration (FDA)–approved intravenous thrombotic therapy for acute ischemic stroke. However, t-PA has a narrow therapeutic window of 4.5 h (Jauch et al., 2013), and delayed t-PA treatment beyond the restricted therapeutic window has detrimental complications such as hemorrhagic transformation (HT), neurotoxicity, and mortality (Wardlaw et al., 2012). Disruption of brain–blood barrier (BBB) integrity is one of the important pathological characteristics of acute ischemic stroke (Barber, 2013).

Matrix metalloproteinases (MMPs) play crucial roles in BBB disruption and brain damage during cerebral ischemia-reperfusion injury (Yang et al., 2007). Activated MMPs disrupt the extracellular matrix (ECM), degrade the neurovascular matrix and tight junction proteins such as claudin-5 and ZO-1, and increase the BBB permeability and brain damage in ischemic stroke (Jickling et al., 2014). The active MMPs also aggravate immunocyte infiltrations and mediate neuroinflammation in hemorrhagic transformation after ischemic stroke (Vandooren et al., 2014). The plasma MMP-9 level has been used as a biomarker to predict hemorrhagic transformation in ischemic stroke with thrombolytic treatment (Ramos-Fernandez et al., 2011). Our recent studies indicate that targeting oxidative stress–induced MMP-9 signaling could be an important therapeutic strategy to reduce BBB disruption and improve stroke outcome (Chen et al., 2015a; Chen et al., 2019). Meanwhile, phosphoinositide-3-kinases (PI3Ks) and their downstream target serine/threonine kinase Akt are important cellular signaling pathways in regulating multiple cellular events, including cell survival and metabolism, cytoskeletal rearrangements, inflammatory responses, and apoptosis (Cantley, 2002). In the process, the phosphorylation of Akt could mediate the activation of glycogen synthase kinase (GSK)-3β. The PI3K/Akt/GSK-3β signaling pathway is one of the crucial signaling pathways and therapeutic targets for neuroprotection against cerebral ischemia injury (Miyawaki et al., 2009; Lu et al., 2011; Dong et al., 2021). Therefore, MMPs and PI3K/Akt/GSK-3β signaling pathways are important therapeutic targets for drug discovery from natural medicinal plants to treat ischemic brain injury.

*Momordica charantia* (MC) is a traditional medicinal plant for the treatment of cardiovascular diseases (Baek et al., 2018). Our previous study indicates that MC polysaccharides could protect against cerebral ischemia/reperfusion injury (Gong et al., 2015; Hu et al., 2020; Ma et al., 2021). Nevertheless, the limited capabilities of crossing the BBB greatly reduce their potential as therapeutic agents. Hence, we seek to develop efficient components from MC with enhanced capacity to cross the BBB for drug development. Plant-derived exosome-like nanoparticles (ELNs) are small vesicles (30∼200 nm) released by multivesicular bodies (Chang et al., 2021; Suharta et al., 2021). Like mammal exosomes, plant-derived ELNs may also transport mRNA, miRNA, bioactive lipids, and proteins into animal cells (Nielsen et al., 2012). The plant-derived ELNs could participate in plant cell-to-cell communication, regulate innate immunity, and attenuate inflammation (Cai et al., 2018; Teng et al., 2018). The nanoparticles (<200 nm) could cross the BBB freely (Brown et al., 2020); hence, ELNs would have great potential for neurological diseases. Recently, ELNs were found in a variety of vegetables and fruits (Mu et al., 2014; Zhuang et al., 2015; Zhang et al., 2016a; Teng et al., 2018). The potential of synthetic gold nanoparticles as nanocarriers for diagnosis and treatment of ischemic stroke has been compromised (Amani et al., 2019). With these unique features, dietary ELNs represent a promising new candidate for translational applications.

In the present study, we successfully isolated and purified exosome-like nanoparticles from *Momordica charantia* and tested the hypothesis that MC-ELNs could cross the BBB and protect BBB integrity by inhibiting MMP-9 activation and attenuating neuronal apoptosis by activating the PI3K/AKT/GSK3β pathway.

## Experimental Section

### Isolation and Characterization of *Momordica charantia*-Exosome-Like Nanoparticles

Fresh *Momordica charantia* samples were purchased in September 2019 from Yunnan Province, China, and authenticated by *Flora Reipubilcae Popularis Sinicae.* MC-ELNs were isolated by continuous differential centrifugation, ultracentrifugation, and then purified by gradient sucrose solution as described previously (Mu et al., 2014). Briefly, *Momordica charantia* was squeezed and continuously centrifuged at 1,000 g for 10 min, 3,000 g for 20 min, and 10,000 g for 40 min at 4°C. The obtained supernatant was ultracentrifuged at 150,000 × g for 90 min (Beckman Optima L-100XP, Beckman, United States), and the pellets were resuspended in phosphate-buffered saline (PBS), transferred to a gradient sucrose solution (8, 30, 45, and 60%), and ultracentrifuged at 150,000 × g for another 90 min. The band between the 30% and 45% sucrose layer was collected and ultracentrifuged at 150,000 × g for 90 min and passed through a 0.22-μm filter to obtain sterile MC-ELNs.

For transmission electron microscope imaging, MC-ELNs were adsorbed on a carbon-coated grid. The grid was stained with 1% uranyl acetate. The absorbed MC-ELNs were examined under a Tecnai G2 Spirit Twin Transmission Electron Microscope (TEM), and the images were recorded with an AMT 2 k CCD camera. The size distribution of the MC-ELNs was analyzed by using Multiple-Laser ZetaView® f-NTA Nanoparticle Tracking Analyzers (Particle Metrix, Germany). Exosome markers including CD54 (Cell signal technology, 4915), CD63 (Proteintech, 25682-1-AP), and TSG101 (Proteintech, 28283-1-AP) were used, and the protein concentration of the MC-ELNs was determined with a BCA commercial kit (Beyotime, P0011) according to the manufacturer’s instructions.

### Animals

Male Sprague–Dawley (SD) rats, weighing 240–270 g, were provided by the Animal Experiment Center of Xuzhou Medical University. The animal experiment was conducted in accordance with the national and institutional guidelines on ethics and biosafety, and the protocol was approved and regulated by the Committee on the Use of Live Animals in Teaching and Research, Xuzhou Medical University. The animals were kept in an environment with controlled temperature and humidity, with a light/dark cycle for 12 h and free access to food and water.

### Middle Cerebral Artery Occlusion Model

The middle cerebral artery occlusion (MCAO) model of focal cerebral ischemia/reperfusion was induced as previously described (Qi et al., 2010). Briefly, the SD rats were anesthetized with inhalation of 4% isoflurane; a silica gel–coated nylon suture was used as an embolus and inserted into the origin of the middle cerebral artery (MCA) *via* the external carotid artery to induce 2 h of ischemia, and then the suture was withdrawn as reperfusion for 24 h. In the sham group, the suture was inserted 5 mm from the incision, and no cerebral ischemia was induced.

### Four-Vessel Occlusion Model

Transient global cerebral ischemia was induced by four-vessel occlusion (4-VO) and modified as previously described (Pulsinelli and Brierley, 1979; Schmidt-Kastner et al., 1989). Briefly, the animals were anesthetized with pentobarbital (40 mg/kg, intraperitoneally), and both vertebral arteries were occluded permanently. Carotid arteries on both sides were occluded with aneurysm clips to induce global cerebral ischemia. After 15 min of occlusion, the aneurysm clips were removed to induce reperfusion for 5 days. Rats with loss of their righting reflex and whose pupils were dilated and unresponsive to light were selected for the experiments.

### 2,3,5-Triphenyltetrazolium Chloride Staining

Triphenyltetrazolium chloride (TTC) staining is commonly applied for the visualization of hypoxic brain tissue and for defining the size of cerebral infarction (Chen et al., 2020a). After being subjected to 2 h of ischemia and 24 h of reperfusion, the animals were killed, and their brains were quickly dissected. The brains were placed at −20°C for 30 min. Then, the brain was cut into 2-mm slices, stained with 2% TTC (A610558, Shanghai Sangon), and incubated at 37°C for 25 min. The brain slices were photographed and preserved in 4% paraformaldehyde (PFA) solution. The relative infarct percentage was calculated by the following formula: Infarct area percentage (%) = (uninfarct hemisphere area-infarct hemisphere uninfarct area)/uninfarct hemisphere area ×100.

### Crystal Violet Staining

Crystal violet staining was performed as previously described (Qi et al., 2013; Chen et al., 2015a). After the 4-VO model was established, the rats were anesthetized and then fixed by 4% PFA perfusion. The brain slices were stained with 0.1% crystal violet (Beyotime, China, C0121). The morphology of the surviving neurons in the hippocampal CA1 area was observed under an Olympus BX51 microscope (Tokyo, Japan). The number of surviving neurons in the area (1 mm) in length was counted.

### Neurobehavioral Score

The neurobehavioral tests were performed 1 day ahead of MCAO and 1 day after MCAO. The experiments were performed by researchers who were blind to the experimental design. The scores included exercise tests, reflex, and sensory tests. The severity score ranges from 0 to 18 according to previous reports (Chen et al., 2020b). Details are included in Supplementary File S1.

### Experimental Designs and MC-ELN Administration

MC-ELNs (200, 400, and 800 μg/kg) were intravenously injected into the rats after 2 h of cerebral ischemia. The optimal dosage of MC-ELNs (800 μg/kg) was chosen from TTC staining of focal ischemia/reperfusion. The rats were randomly divided into three groups: sham (labeled as control), MCAO 2 h/reperfusion 24 h (labeled as MCAO), and MCAO 2 h/reperfusion 24 h plus 800 μg/kg MC-ELNs (labeled as MC-ELNs).

### 1,1′-Dioctadecyl-3,3,3′,3′-Tetramethylindocarbocyanine Perchlorate (Dil)-Labeling of MC-ELNs

Dil dye was used to label ELNs as previously described (Elashiry et al., 2021). MC-ELNs were incubated with Dil (C1036, Beyotime, China) at 37°C in the dark for 15 min. After centrifugation at 150,000 g for 90 min, the pellet was resuspended and washed with PBS twice to remove the free Dil dye. The pellets were resuspended to obtain Dil-labeled MC-ELNs.

### Blood–Brain Barrier Permeability Detection

Evans blue (EB) was used to analyze BBB permeability as previously described (Naderi et al., 2015). In brief, the rats were anesthetized and injected intravenously with 2% EB (Beijing Yinuokai Technology Co., Ltd., 3 ml/kg, 1 h before sacrifice). The rats were perfused with PBS to remove circulating EB. Then, the brain was harvested and divided into two consecutive millimeter-thick coronal slices, and the digital photos of EB extravasation were captured. The tissue was divided into an ischemia side and non-ischemia side, weighed, and stored at −80°C until use. The tissue was homogenized in cold PBS and further homogenized with the same volume of 50% trichloroacetic acid (Beijing Yinuokai, China). After being centrifuged at 15,000 rpm, the supernatant was collected. The optical density value was measured in a microplate reader (Bio-Rad, Hercules, CA, United States). The amount of EB dye was quantified as microgram per gram of brain tissue.

### Western Blotting

The total protein was extracted with RIPA lysis buffer (Merck, 20–188). Then, the protein was separated by sodium dodecyl sulfate–polyacrylamide gel electrophoresis and transferred to polyvinylidene fluoride membranes. The membrane was blocked with 5% non-fat milk at room temperature, followed by incubation with the following primary antibodies: anti CD54 (Proteintech, 10831-1-AP, 1:1000), anti CD63 (Proteintech, 25682-1-AP, 1:1000), anti-TSG101 (Proteintech, 28283-1-AP, 1:1000), anti–ZO-1 (Abcam, ab190085, 1:1000), anti–MMP-9 (Abcam, ab76003, 1:1000), anti–claudin-5 (Abcam, ab172968, 1:1000), anti–cleaved caspases-3 (9661, CST, 1:1000), anti–Bcl-2 (Abcam, ab196495, 1:1000), and anti-Bax (Abcam, ab32503, 1:1000) overnight at 4°C. The secondary antibody was incubated at room temperature for 1 h. The membrane was developed with the ECL Western Blotting Substrate (Thermo Fisher, United States) and captured with ChemiDoc Touch (Bio-rad, United States), and the band was processed and analyzed by ImageJ software.

### Small RNA-Seq of MC-ELNs and Data Analysis

Omicsolution (Shanghai, China) performed small RNA-sequencing of MC-ELNs (*n* = 3). The MC-ELN RNA was isolated using the TRIzol reagent (Thermo Fischer Scientific). The small-RNA was obtained by PAGE electrophoresis for library preparation and sequencing. Small RNA libraries were created with the MGIEasy Small RNA Library Preparation Kit V2.0 (MGI, Shenzhen, China) according to the manufacturer’s protocol. The final purified library was quantified with a small RNA chip (Agilent 2100 Bioanalyzer) and the StepOnePlus real-time PCR assay (ABI, United States), followed by a combinatorial probe-anchor synthesis (cPAS) using a BGISEQ-500RS High-throughput sequencing set (MGI, Shenzhen, China) according to the manufacturer’s instructions. The small RNA-sequencing data analysis is performed using a Linux local system. Next, ncRNAs (miRNAs, tRNAs, rRNAs, lincRNAs, piRNAs, and snoRNAs), antisense transcripts, coding genes, and repeat elements (LTR, LINE, SINE, and tandem repeats) were determined. The sequence reads were mapped to known plant mature miRNAs in miRBase22.0, and mapping statistics were generated.

### MiRNA Target Gene Analysis

The miRNA target gene analysis was performed using miRanda and PITA, the web tools that identify enriched pathways targeted by selected miRNAs in rats (Wu et al., 2012). The miRID was based on miRbase version 22.0. Then, we took the intersection of the target gene prediction results obtained by the two methods as the miRNA target gene prediction result.

### Validation of Selected miRNA Using Quantitative Real-Time PCR

In the annotated 81 miRNAs from RNA-seq data, miR5266 was predicted to contribute to binding with MMP-9. The total RNA was isolated from three biological replicates of MC-ELNs using the TRIzol reagent. An miRNA First-Strand cDNA Synthesis Kit (Sangon, Shanghai, China) was used for converting specific miRNA into cDNA. A SYBR Premix Ex Taq II PCR kit (Takara) and PCR assay primer mix (miR5266 forward primer: TAT​ACG​CGG​GGG​ACG​GAC, reverse primer: AGT​GCA​GGG​TCC​GAG​GTA​TT, reverse transcription primer: GTC​GTA​TCC​AGT​GCA​GGG​TCC​GAG​GTA​TTC​GCA​CTG​GAT​ACG​ACC​GCC​CA) were used to measure the level of miRNA5266.

### Dual-Luciferase Reporter Assay

The wild-type (WT) and mutant of 3′-UTR fragments of MMP-9 containing four putative miR5266 binding sites were synthesized by Genechem (Shanghai, China) and were cloned into a GV272 reporter (SV40-firefly_Luciferase-MCS). The detailed sequence of the WT and mutant of 3′-UTR fragments of MMP-9 is given in Supplementary File S2. HEK293T cells were cultured in DMEM supplemented with 10% FBS, 1% glutamax, and 1% penicillin and streptomycin (Gibco, United States). For the dual-luciferase reporter assay, HEK293T cells were co-transfected with miR5266 mimic, GV272-mmp-9-WT or GV272-mmp-9-Mut reporter plasmids, and CV045 plasmid (TK promoter-Renilla_Luciferase). Luciferase activity was measured with the Dual-Glo™ Luciferase Assay System (E2920, Promega) using a GloMax 20/20 luminometer (Promega) according to the manufacturer’s instruction.

### TUNEL Staining

The cryosections (slice thickness 20 µm) were prepared as previously described (Yang et al., 1979). TUNEL staining was performed in accordance with the instructions in the TUNEL detection kit (Dalian Meilun Biotechnology Co., Ltd.). Then, 4′,6-diamidino-2-phenylindole (DAPI) staining was performed for 20 min, and the nucleus was observed under a fluorescence microscope (Tokyo, Japan).

### Cell Culture and Oxygen-Glucose Deprivation/Reoxygenation Model

The mouse hippocampal neuronal HT22 cells were purchased from ATCC. They were cultured in a complete medium (high glucose DMEM medium, 10% fetal bovine serum, and 1% penicillin and streptomycin). To mimic cerebral ischemia/reperfusion injury *in vitro*, HT22 cells were exposed to oxygen-glucose deprivation for 9 h and reoxygenation (OGD/R) according to a previous protocol (Jiang et al., 2000). For OGD/R, the medium was changed to glucose-free DMEM supplemented with 10% fetal bovine serum and 1% penicillin and streptomycin and cultured in an incubator perfused with O_2_/CO_2_ (1.2%/5%) tri-gas. After 9 h of OGD, the cells were replaced with the complete medium and cultured in the incubator perfused with 21% O_2_/5%CO_2_ for 24 h to induce reoxygenation. These cell experiments were also divided into three groups: control, oxygen-glucose deprivation/reoxygenation (labeled as OGD), and OGD plus 10 μg/ml MC-ELNs (labeled as MC-ELNs, treated before and after 9 h of OGD).

### Flow Cytometry

HT22 cells were inoculated at 4 × 10^5^ cells/well and subjected to OGD/R. MC-ELNs were applied to HT22 cells before and after OGD. These cells were digested with EDTA-free trypsin, mixed, and suspended with a Binding Buffer and Annexin V-PE solution (KeyGen, China). Before testing, 7-AAD reagents were added and detected by flow cytometry (BD Biosciences), and data were analyzed with FlowJo software (FlowJo, Ashland, OR, United States).

### Data Analysis

Data were expressed as mean ± standard deviation (SD), and statistical analysis was performed by GraphPad prism 8.0.2 software. The data of Dil-labeled MC-ELNs and dual-luciferase assay were analyzed by two-way analysis of variance (two-way ANOVA). The other experiments were all analyzed by one-way ANOVA followed by Dunnett’s t post-hoc test. *p* < 0.05 was defined as significant differences.

## Results

### Isolation and Characterization of MC-ELNs

**FIGURE 1:**
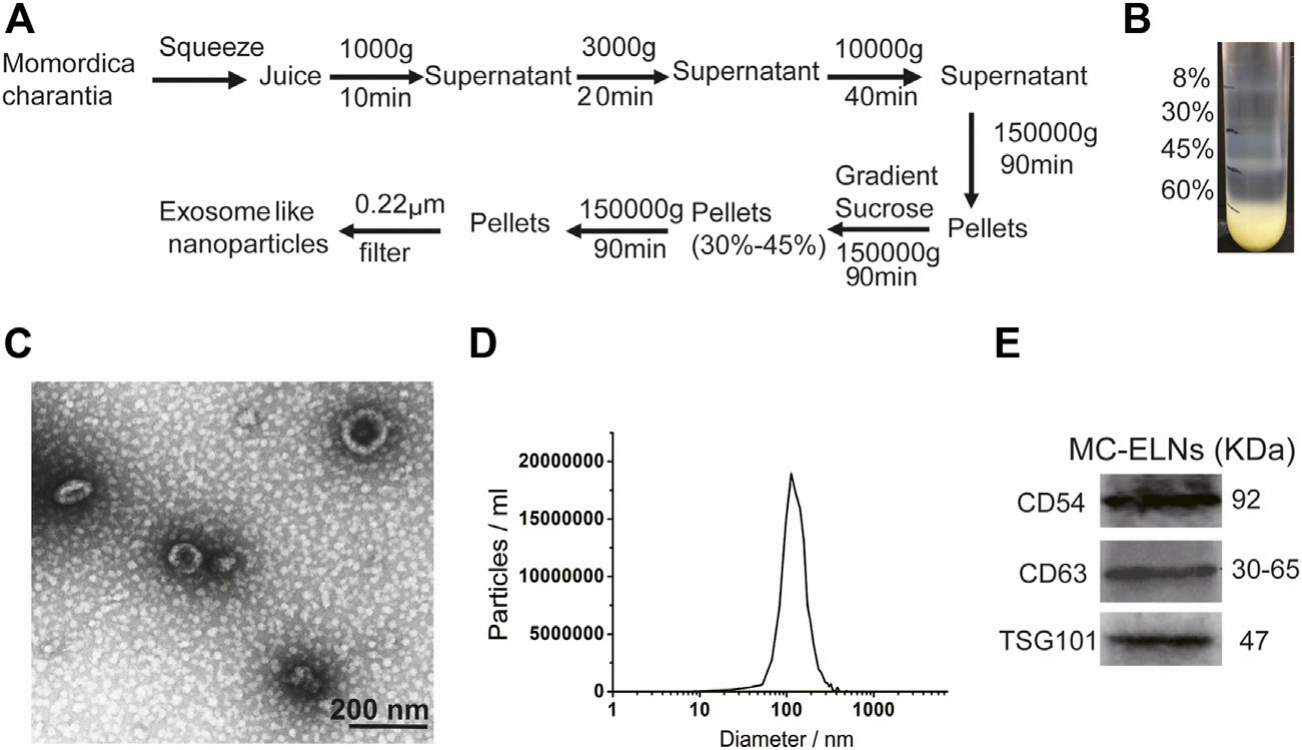
Isolation and characterization of MC-ELNs. **(A)** Schematic representation of ELN isolation from MC. **(B)** MC-ELNs were purified by sucrose density gradient (8%/30%/45%/60%) under ultracentrifugation, and the band between 30% and 45% was harvested and defined as MC-ELNs. **(C)** Representative MC-ELN images obtained by TEM, scale bar = 200 nm. **(D)** MC-ELN tracking analysis. **(E)** Representative immunoblotting images of exosomal markers in MC-ELNs.

The MC-ELNs were separated by differential centrifugation and ultracentrifugation and purified by 8, 30, 45, and 60% gradient sucrose, and the band between 30% and 45% was collected as MC-ELNs (Figures 1A,B), and the MC-ELNs were characterized by transmission electron microscopy, nanoparticle tracking analysis, and Western blot analysis. The MC-ELNs showed typical spherical or cup-shaped morphology under transmission electron microscopy (Figure 1C). Nanoparticle tracking analysis showed that the mean particle diameter of the MC-ELNs was 131.6 nm (Figure 1D). Western blotting analysis revealed the expressions of exosome markers including CD54, CD63, and TSG101 in the MC-ELNs (Figure 1E).

### MC-ELNs Alleviated Cerebral Ischemia, Promoted Hippocampus CA1 Neuron Survival, and Improved Neurological Functions in Cerebral I/R Injury

**FIGURE 2:**
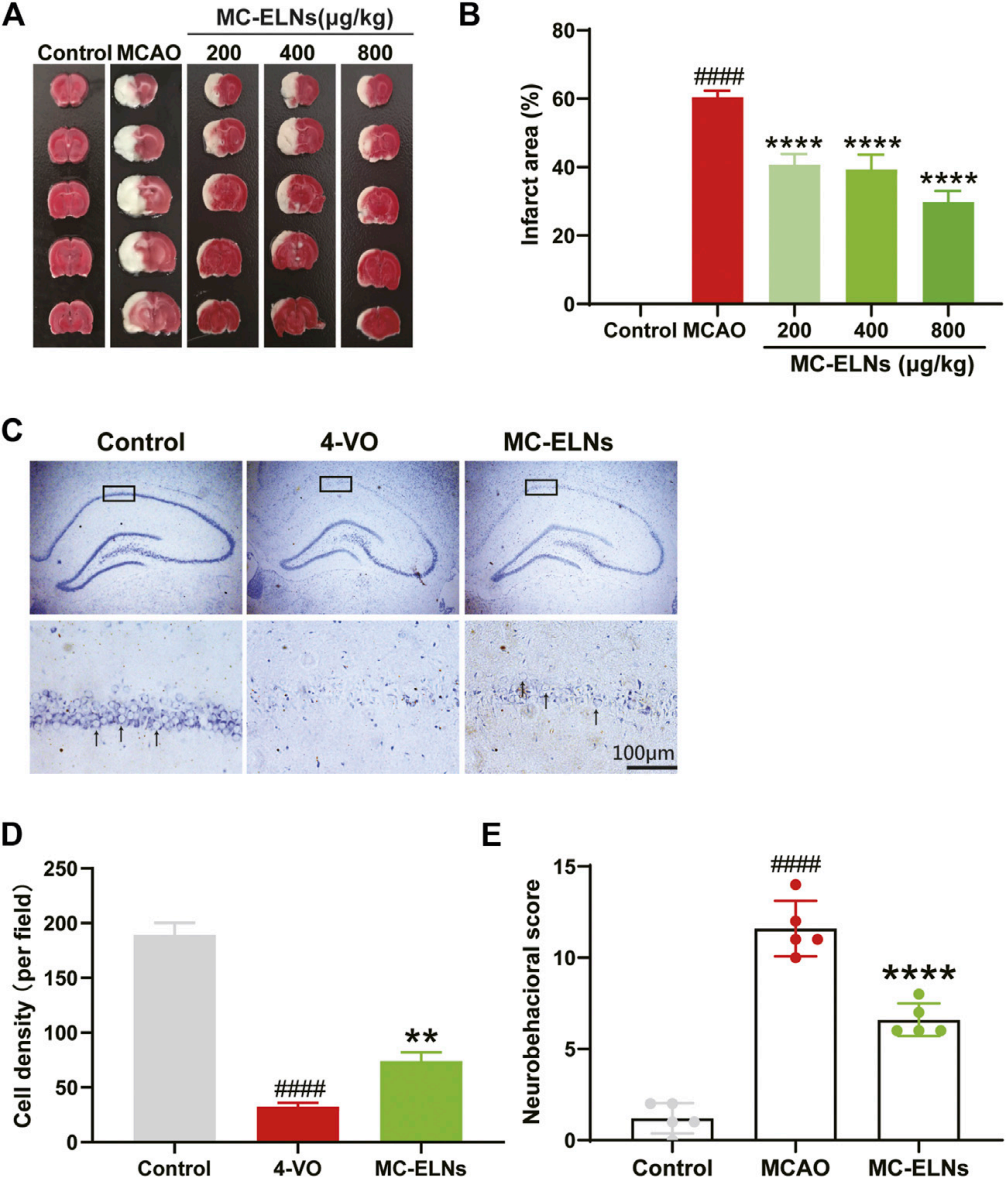
MC-ELNs alleviate cerebral ischemia, promote hippocampus CA1 neuronal survival, and improve neurological function in transient cerebral ischemic rats. Sham control operation was used as the control group. The MCAO group was subjected to 2 h of MCAO ischemia followed by 24 h of reperfusion which was received through vehicle treatment. In the MC-ELN group, MCAO rats received intravenous injection of MC-ELNs (200, 400, and 800 μg/kg) after being exposed to 2 h of MCAO cerebral ischemia followed by 24 h of reperfusion. **(A)** Representative TTC staining images show the cerebral infarct area in MCAO rats treated with MC-ELNs of different concentrations. **(B)** Quantitative analysis of the percentage of the infarct area in each group, ^*####*^ *p* < 0.0001, *vs*. control, ****p < 0.0001, *vs*. MCAO (*n* = 3). **(C)** Representative images show the cell density of hippocampus CA1 neurons by crystal violet staining. Black arrows indicate the living neurons. **(D)** Quantitative analysis of the number of neurons in the CA1 region of the hippocampus, ^*####*^ *p* < 0.0001, *vs*. control, **p < 0.01, *vs*. 4-VO (*n* = 3). **(E)** mNSS neurological severity scores showed that MC-ELN treatment could improve neurological deficits in MCAO ischemic rats, ****p < 0.0001, *vs*. control, and ^*####*^ *p* < 0.0001, *vs*. MCAO (*n* = 5).

We then investigated whether MC-ELNs could alleviate cerebral ischemic/reperfusion (I/R) injury. TTC staining showed that injection of MC-ELNs *via* the tail vein (200, 400, and 800 μg/kg) significantly reduced infarct sizes in I/R rats in a concentration-dependent manner (Figure 2A). The infarct area percentage of the MCAO group was about 59.30 ± 2.78%. Treatments of MC-ELNs at the dosages of 200, 400, and 800 μg/kg were 47.37 ± 14.66%, 42.60 ± 8.45%, and 29.77 ± 3.26%, respectively (*p* < 0.0001, *p* < 0.0001, and *p* < 0.0001, Figure 2B). The optimum dosage was 800 μg/kg MC-ELNs. Thus, we chose 800 μg/kg MC-ELNs for the following experiments. By performing crystal violet staining, we analyzed neuronal survival in the hippocampal CA1 area of the rats subjected to the cerebral four-vessel occlusion (4-VO) model. After 15 min of cerebral ischemia plus 5 days of reperfusion, the rats revealed a significantly decreased number of live neurons in the CA1 area. MC-ELN treatment significantly increased the number of live neurons in the CA1 area (*p* < 0.01, Figures 2C,D). We then evaluated the neurological damage by using the modified neurological severity score (mNSS). Compared with the MCAO group, MC-ELN treatment had significantly decreased the mNSS score, indicating the improvement of neurological functions (*p* < 0.0001, Figure 2E). Taken together, MC-ELNs have neuroprotective effects against cerebral I/R injury.

### MC-ELNs Could Cross the Blood–Brain Barrier and Reach Infarct Area in MCAO Rats

**FIGURE 3:**
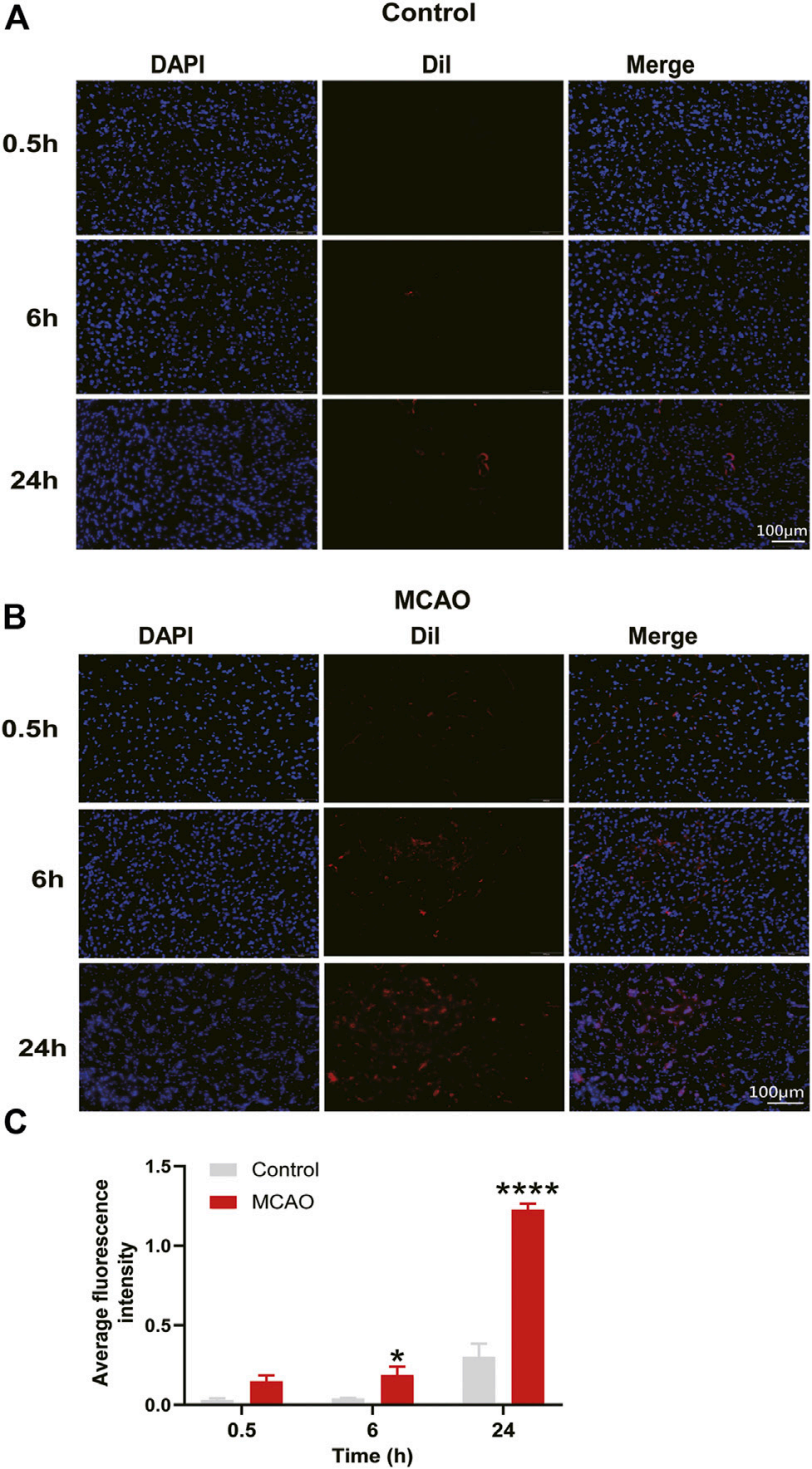
MC-ELNs have the capacity of crossing the BBB and accumulate in the infarct area of ischemic brains. **(A,B)** Representative immunofluorescent images of the ipsilateral striatum at indicated time points after injection of Dil-labeled MC-ELNs (800 μg/kg) in control and MCAO rats; red fluorescence represents Dil and blue indicates DAPI. **(C)** Fluorescence intensity of Dil was analyzed at each time point in control and MCAO groups. *p < 0.05, ****p < 0.0001, *vs*. control (*n* = 3).

To verify whether MC-ELNs could pass through the BBB, we used the dye Dil to label MC-ELNs. After 2 h (h) of cerebral ischemia in rats, we injected Dil-labeled MC-ELNs intravenously followed by reperfusion for 0.5, 6, and 24 h. The fluorescence intensity analysis showed that although Dil increased with time in the control group, compared with the control group, the intensity of Dil in the striatum of the MCAO group was slightly increased at 6 h (*p* < 0.05) but remarkably increased at 24 h (*p* < 0.0001, Figure 3). It hinted that MC-ELNs can travel across the BBB freely after cerebral I/R injury, as BBB permeability increased, and MC-ELNs were easily able to penetrate the BBB into the brain.

### MC-ELNs Inhibited MMP-9, Preserved Tight Junction Proteins, and Protected the Blood–Brain Barrier Integrity in MCAO Rats

**FIGURE 4:**
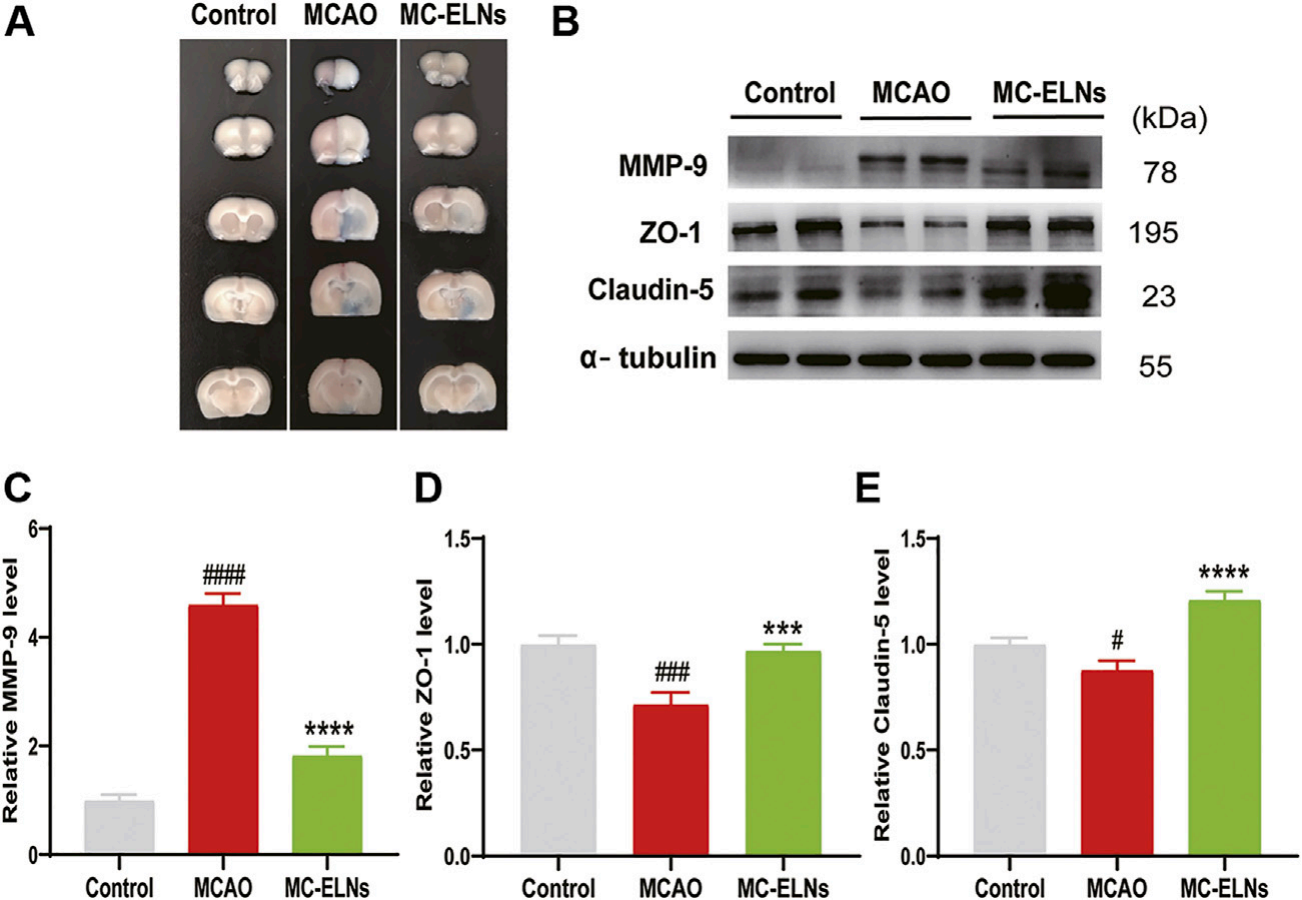
MC-ELN treatment inhibits MMP-9 expression, reserves the expression of claudin-5 and ZO-1, and protects the BBB integrity in transient cerebral ischemic rats. The BBB permeability was identified by Evans blue test. The expression of MMP-9, claudin-5, and ZO-1 was detected with Western blot analysis. **(A)** Representative images show the EB leakage in the brain sessions of control, MCAO, and MC-ELN groups. **(B–E)** Immunoblotting and statistical analysis of the expression of MMP-9, claudin-5, and ZO-1 in each group. α-tubulin was used as the internal control. ^*#*^ *p* < 0.05, ^*###*^ *p* < 0.001, and ^*####*^ *p* < 0.0001, *vs*. control, ***p < 0.001, ****p < 0.0001, *vs*. MCAO (*n* = 3).

We then performed EB leakage experiments to evaluate the effects of MC-ELNs on BBB integrity. After MCAO, the EB leakage was significantly increased; however, 800 μg/kg MC-ELNs significantly reduced the EB leakage in ischemic brains, indicating the protection of the BBB integrity, Figure 4A. It is well known that MMP-9 plays crucial roles in BBB disruption by degrading the neurovascular matrix and tight junction proteins such as claudin-5 and ZO-1 in ischemic brain injury (Jickling et al., 2014). Thus, we evaluated the effect of MC-ELNs on the expressions of MMP-9, claudin-5, and ZO-1 in ischemic brains. As expected, the rats subjected to MCAO had significantly higher expression levels of MMP-9 but lower expression of claudin-5 and ZO-1 in the brain than in the control group (*p* < 0.0001, *p* < 0.01, and *p* < 0.05, Figures 4B–E). However, MC-ELN treatment significantly reduced the level of MMP-9 but increased the levels of claudin-5 and ZO-1 (*p* < 0.0001, *p* < 0.001, and *p* < 0.0001, Figures 4B–E). These results indicate that MC-ELNs could protect the BBB integrity by inhibiting MMP-9 and preserving tight junction proteins in ischemic brain injury.

### MiR5266 Derived From MC-ELNs Bound With the 3′UTR of MMP-9

**FIGURE 5:**
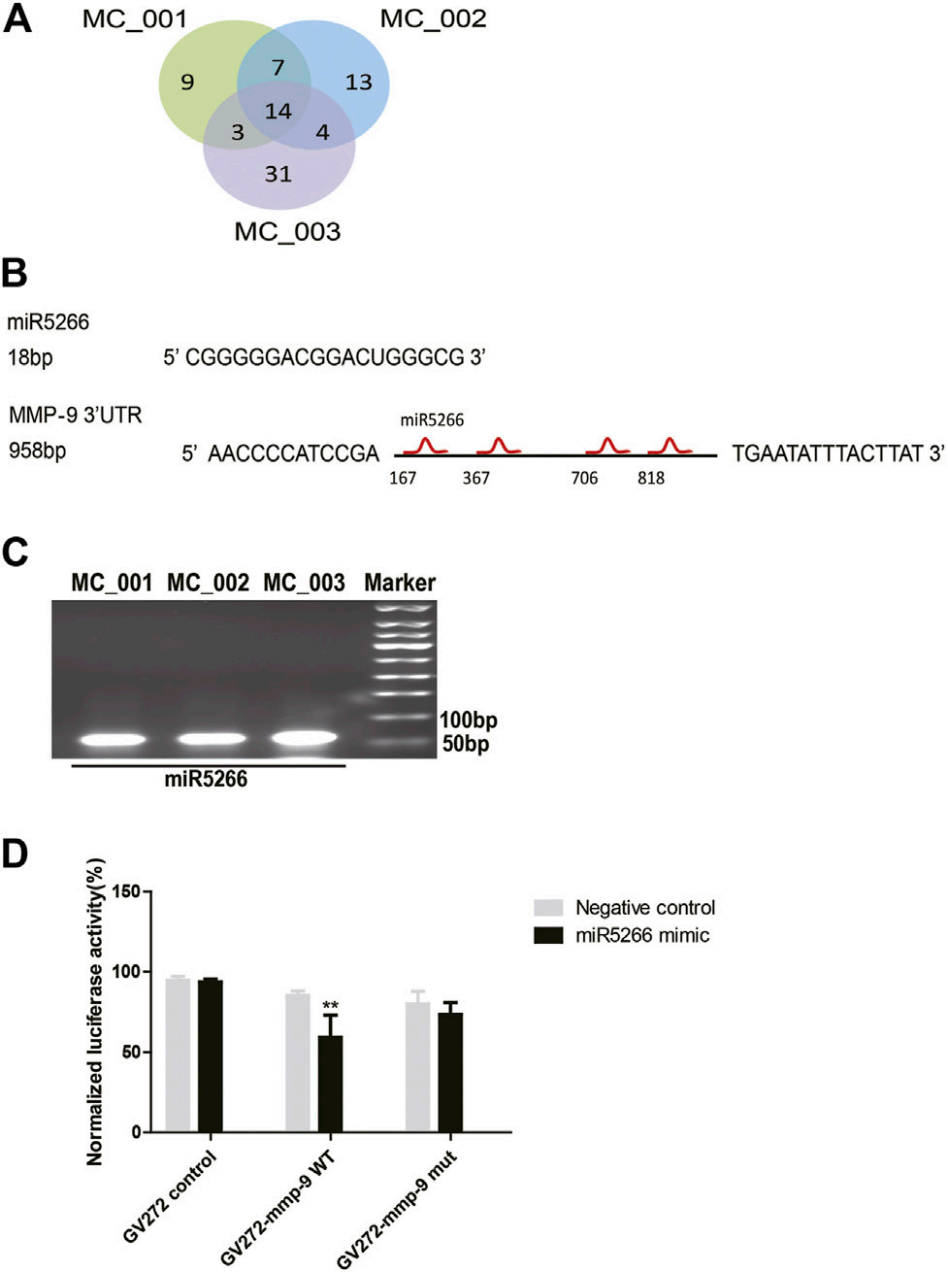
MiR5266 derived from MC-ELNs bound with the 3′UTR of MMP-9. **(A)** Venn diagram shows miRNA tag distribution among three batches of MC-ELN samples. **(B)** Sequence of miR5266, miR6250, and four putative sites where miR5266 binds to MMP-9 3′UTR. **(C)** Representative image of the expression of miR5266 in MC-ELNs by qPCR. **(D)** Dual-luciferase reporter gene assay confirmed that MMP-9 was the target gene of miR5266, **p < 0.01, *vs*. negative control (*n* = 3).

To elucidate the underlying neuroprotective mechanisms, we performed small RNA-seq analysis to clarify the small RNA components in MC-ELNs. We detected miRNA, rRNA, snRNA, snoRNA, and tRNA in three MC-ELN samples. Except for unannotated tags, miRNAs are the most abundant small RNAs (Supplementary Tables S1, S2). We identified 81 miRNAs in MC-ELNs by referring mature plant miRNA in miRbase 22.0 (Figure 5A, Supplementary Tables S3). Among the 14 miRNAs (Figure 5A) that were expressed in all the three MC-ELN samples, miR5266 with relatively high abundance (Supplementary Table S3) could bind with the 3′UTR of MMP-9 by target gene prediction analysis (Figure 5B). The presence of miR5266 in MC-ELNs was further confirmed by RT-qPCR analysis (Figure 5C). Hence, we chose miR-5266 for further investigation. Using a well-developed miRNA and target-finding algorithm (https://www.targetscan.org/), we identified four potential binding sites of miR5266 with the 3′UTR of the MMP-9 gene (Supplementary File S2). Furthermore, we constructed two luciferase reporter plasmids containing a fragment sequence of the wild-type (WT) or mutant (Mut) binding site of MMP-9. The dual-luciferase reporter assay revealed that miR5266 reduced the luciferase activity in MMP-9 WT but not the MMP-9 Mut-transfected HEK293T cells (*p* < 0.01, Figure 5D). These results suggested that miR5266 in MC-ELNs could specifically inhibit MMP-9 by binding to its coding sequences within the 3′-UTR region.

### MC-ELNs Reduced Apoptosis in MCAO Rats

**FIGURE 6:**
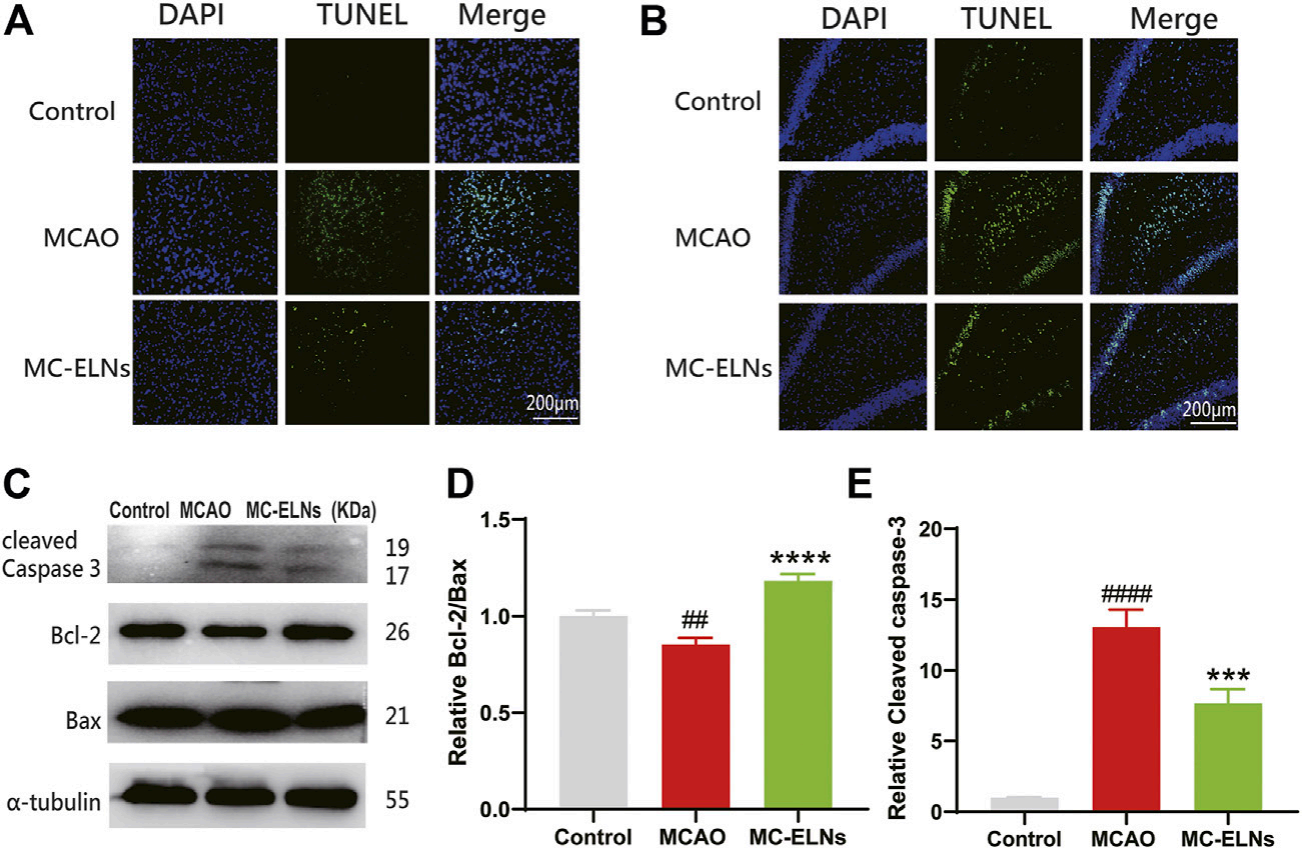
MC-ELN treatment reduced neuronal apoptosis in transient cerebral ischemic rats. **(A)** Representative TUNEL staining images of the ipsilateral striatum in each group. **(B)** Representative TUNEL staining images of the hippocampal DG area in each group. **(C–E)** Immunoblotting and statistical analysis of cleaved caspase-3, Bcl-2, and Bax expression in brain tissue. ^*##*^ *p* < 0.05, ^*####*^ *p* < 0.0001, *vs*. control, ***p < 0.001, ****p < 0.0001, *vs*. MCAO (*n* = 3).

We then investigated the effects of MC-ELNs on apoptotic cell death in MCAO ischemic brains. The results revealed that the rats with MCAO had remarkably enhanced TUNEL-positive cells in the striatum and hippocampus, indicating that there were more apoptotic cells after MCAO. MC-ELN treatment significantly reduced apoptosis in the striatum and hippocampus of MCAO ischemic brains (Figures 6A,B). Furthermore, MC-ELN treatment also significantly increased the ratio of Bcl-2 to Bax in MCAO ischemic brains (*p* < 0.0001, *n* = 3) (Figures 6C,D). In addition, MC-ELNs significantly inhibited the expression of cleaved caspase-3 in MCAO ischemic brains (*p* < 0.001, *n* = 3) (Figures 6C,E). These results suggest that MC-ELNs have the capability to reduce apoptotic cell death in MCAO ischemic brains.

### MC-ELNs Induced Phosphorylation of AKT and GSK3β and Inhibited Apoptosis After Being Exposed to OGD/R

**FIGURE 7:**
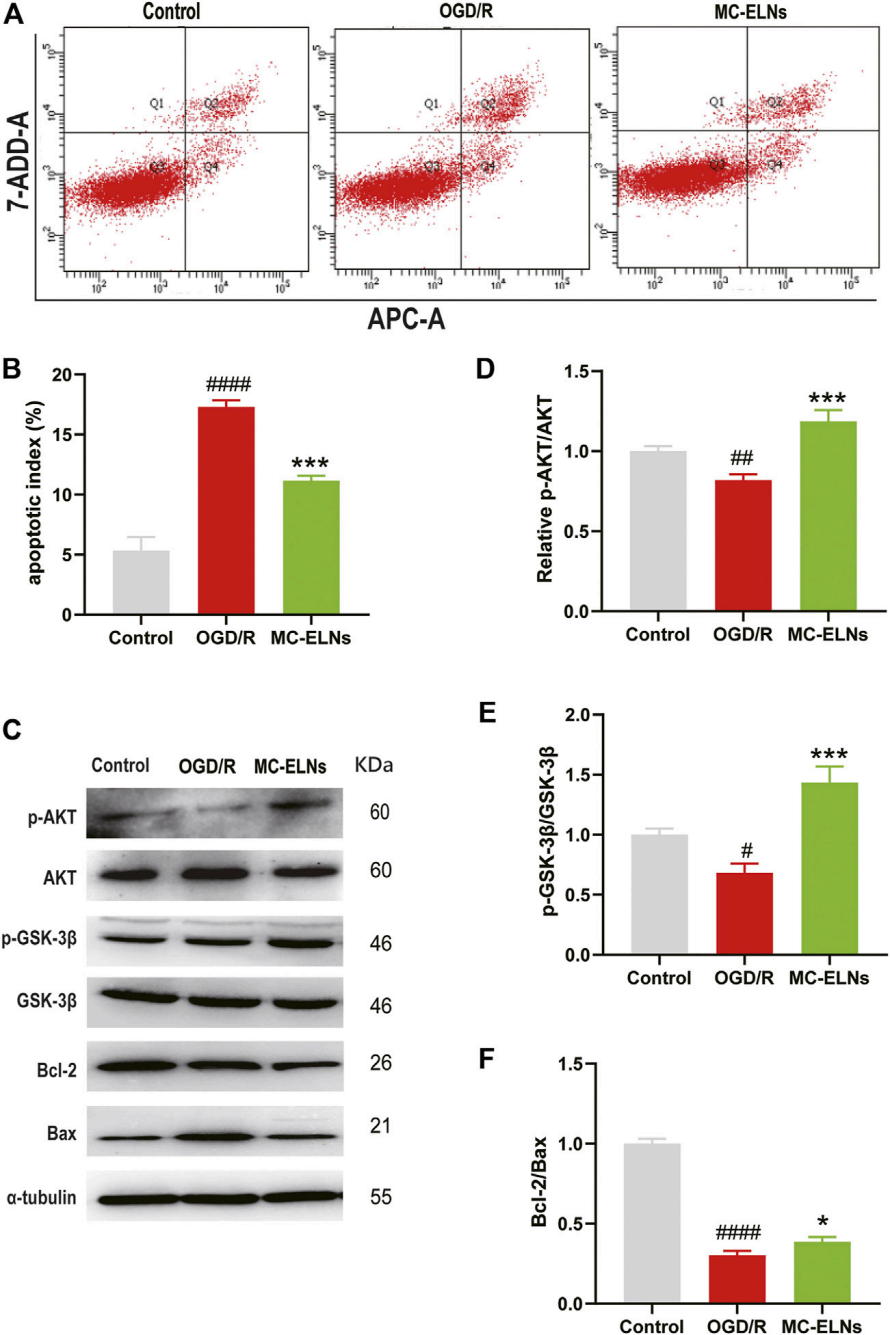
MC-ELN treatment induced the phosphorylation of AKT and GSK3β and inhibited apoptosis in OGD/R-treated HT22 cells. Control, the HT22 cells were exposed to normoxic and normal culture conditions; OGD/R, the HT22 cells were exposed to 9 h OGD plus 24 h reoxygenation with vehicle treatment. MC-ELNs, the HT22 cells were exposed to 9 h OGD plus 24 h reoxygenation with MC-ELN treatment (10 μg/ml). **(A)** Flow cytometry detection for the effects of MC-ELNs on OGD/R-induced apoptosis of HT22 cells. **(B)** Statistical analysis on the rates of apoptotic cells, ^*####*^ *p* < 0.0001, *vs*. control, ***p < 0.001, *vs*. OGD/R (*n* = 3). **(C)** Immunoblotting images for the expression of the phosphorylated AKT and GSK-3β, Bcl-2, and Bax in the HT22 cells. **(D–F)** Statistical analysis on the expression of the phosphorylated AKT and GSK-3β, and the ratio of Bcl-2 to Bax in HT22 cells. ^*#*^ *p* < 0.05, ^##^ *p* < 0.01, ^*####*^ *p* < 0.0001 *vs*. control, *p < 0.05, ***p < 0.001, *vs*. OGD/R (*n* = 3).

The PI3K/Akt/GSK-3β signaling pathway plays crucial roles in neuroprotection against cerebral ischemia injury (Miyawaki et al., 2009; Lu et al., 2011). To explore the underlying neuroprotective mechanisms of MC-ELNs, we detected apoptotic rates, the phosphorylation of AKT and GSK3β, and the ratio of Bcl-2 to Bax in cultured mouse hippocampus neuronal cell line HT22 cells after being exposed to OGD/R. The OGD/R treatment had significantly higher apoptotic rates than the control group (OGD/R 17.30 ± 0.56%, *vs.* control 5.47 ± 0.46%, *p* < 0.001, *n* = 3) (Figures 7A,B). The MC-ELN group had significantly lower apoptotic rates than the OGD/R vehicle group (MC-ELNs 11.17 ± 0.40%, *vs*. OGD/R 17.30 ± 0.56%, *p* < 0.001, *n* = 3) (Figures 7A,B). Immunoblotting studies revealed that the OGD/R group had significantly lower expression of phosphorylation of AKT whose effects were reserved by the treatment of MC-ELNs (*p* < 0.001, Figures 7C,D). Meanwhile, the OGD/R group had a lower expression level of GSK-3β phosphorylation and a lower ratio of Bcl-2/Bax than the control group. The MC-ELN administration had significantly increased the phosphorylation level of GSK-3β and the ratio of Bcl-2 to Bax (*p* < 0.001, *p* < 0.05, Figures 7C,E,F). These results indicate that MC-ELNs exert neuroprotective effects by activating the AKT/GSK-3β signaling pathway.

## Discussion

At present, the research on plant ELNs in the treatment of ischemic stroke is extremely scarce. However, because of the availability and standardized extraction process of plant ELNs, their application potential in the development of new drugs for diseases such as ischemic stroke is promising. In this study, we characterized the nanoparticles named MC-ELNs from *Momordica charantia* and reported its neuroprotective effects against cerebral I/R injury by inhibiting MMP-9 and activating the AKT/GSK-3β signaling pathway (graphical abstract). Our results showed that MC-ELNs could penetrate the BBB into the infarct area, protect BBB integrity, and alleviate cerebral I/R injury. MC-ELN–derived miR-5266 might contribute to the bioactivity of inhibiting MMP-9 expression. Meanwhile, MC-ELNs could activate the AKT/GSK-3β signaling pathway and inhibit neuronal apoptosis. Thus, the MC-ELNs could be a novel drug candidate for the treatment of ischemic stroke.

Mammal extracellular vesicles (EVs) contain two forms, micro-vesicles (200–1000 nm) and exosomes (50–100 nm) (Chang et al., 2021). Plant EVs also differ in size. Exosomes deliver cargos into recipient cells including nucleic acid, lipid, and protein for cell–cell communication. Recently, different exosomes obtained from animal cells were reported to have neuroprotective effects, and the sources of animal cells included mesenchymal stem cells (MSCs) (Han et al., 2018), embryonic stem cells (ESCs) (Kalani et al., 2016), neural stem cells (NSCs) (Spellicy et al., 2020), mononuclear cells (MNCs) (Beer et al., 2016), and astrocytes (Pei et al., 2019). However, the use of animal-derived exosomes is still controversial because of ethical and legal consideration. Moreover, it is also difficult to control the purity and quality of exosomes because of the limitation of the conditioned medium and different extraction requirements (Zhang et al., 2016b). The extraction of plant exosome-like nanoparticles (or extracellular vesicles) is relatively simple and uniform. A set of standard methods for extracting plant exosome-like nanoparticles have been established. Therefore, it is very economical and effective to develop plant-derived exosome-like nanoparticles. The exosome-like nanoparticles or nanovesicles from ginger, lemon citrus, grape, and dried nuts have shown anti-inflammatory and antioxidant properties and show bioactivities for the treatments of colitis (Zhang et al., 2016a), tumor (Raimondo et al., 2018), and liver damage (Zhuang et al., 2015). For instance, ginger ELN-derived miRNAs targeting the SARS-CoV-2 genome has the potential to be developed as an alternative therapy (Kalarikkal and Sundaram, 2021; Teng et al., 2021). Ginger ELNs are preferentially taken up by Lactobacillaceae in a GELN lipid-dependent manner and contain microRNAs that target various genes in Lactobacillus rhamnosus (LGG) (Teng et al., 2018). Plants send small RNAs in extracellular vesicles to fungal pathogens to silence virulence genes (Cai et al., 2018). Green tea leaves nanotherapeutics could prevent and alleviate colitis-related cancer (Zu et al., 2021). So far, no plant ELN has been reported to treat neurological diseases, especially ischemic stroke. MC is a medicinal plant with anti-inflammatory and antioxidant effects (Nerurkar et al., 2011). In the present study, we have purified small vesicles with a diameter of about 131.6 nm from MC. There are various reports which chose different purification bands as ELNs; apparently, each band contains the bioactive components (Zu et al., 2021); however, most reports chose the band between 30% and 45%; hence, we also chose the 30%–45% band. The other controversial issue is whether we can use CD54, CD63, and TSG101 as plant ELN markers. At present, there have been no specific markers for plant ELNs because of technical obstacles, and these markers are widely accepted now.

We investigated the neuroprotective effects of MC-ELNs against cerebral I/R injury in the two rat models of cerebral I/R injury, MCAO (Figures 2A,B,E) and the 4-VO model (Figures 2C,D). For nanoparticles less than 200 nm, it is relatively easy to cross the BBB freely despite the shape and material (Brown et al., 2020). Thus, we first observed the distributions of Dil-labeled MC-ELNs in both sham (control) and MCAO rat brains. The results showed that the Dil-labeled MC-ELNs were identified in the brains of both sham and ischemic brains, particularly in the infarct area of post-ischemic brains (Figures 3A–C). The accumulation of Dil-labeled MC-ELNs in ischemic brains indicates the BBB permeability of the MC-ELNs and its potential for ischemic stroke treatment.

During ischemic stroke, a large amount of plasmin, hydrolase, and free radicals are released, subsequently activating MMP-9 and aggravating ischemic brain injury (Chen et al., 2006; Sarfo et al., 2018). Activated MMP-9 degrades the extracellular matrix and destroys BBB integrity, causing vasogenic brain edema. In our previous studies, we reported that several natural compounds, including calycosin-7-O-β-D-glucoside, baicalin, *Momordica charantia* polysaccharide (MCP), chlorogenic acid, lutein, and lycopene, could target MMP signaling to protect against cerebral ischemia-reperfusion injury (Chen et al., 2015b; Gong et al., 2015; Chen et al., 2018). In the present study, we found that the treatment of MC-ELNs downregulated the expression of MMP-9 and upregulated the expressions of tight junction proteins claudin-5 and ZO-1 in cerebral I/R injury (Figures 4B–E. Furthermore, MC-ELNs significantly decreased infarct sizes and mNSS scores and protected the BBB integrity in rats after being subjected to cerebral ischemia and reperfusion (Figures 2A,B,E; Figures 4A,B), indicating that MC-ELNs have neuroprotective effects against cerebral I/R injury.

MicroRNAs (miRNAs) are small (18–24 nt) noncoding RNAs impacting fundamental biological processes such as proliferation, differentiation, immune responses, and so on (Chen et al., 2006; Hirschberger et al., 2018). Accumulating evidence supports that miRNA-rich micro-vesicles can be transferred into recipient cells and play an important role in intercellular communication (Redis et al., 2012). The first direct proof is that plant-derived miR168a can enter the serum and bind to low-density-lipoprotein receptor adapter protein 1 (LDLRAP1) mRNA to decrease LDL levels (Zhang et al., 2012). In the present study, we performed small RNA-seq analysis and found that MC-ELNs had abundant miRNA (81 miRNAs were annotated) (Supplementary Tables S1, S2, S3). miR5266 was the highest expressed among the 14 miRNAs that were identified in all the three MC-ELN samples (Figure 5A, Supplementary Table S3). There were no reports of the biological function of miR5266 in animal/human disease models. Here, the target gene prediction analysis and dual-luciferase reporter assay showed that miR5266 could bind with MMP-9 3′UTR and inhibit the expression of MMP-9 (Figures 5A–D). Thus, miR5266 could be one of the miRNAs contributing to the bioactivities of MC-ELNs against cerebral I/R injury. It is valuable to further identify other miRNAs with neuroprotective effects.

It is well known that the PI3K/Akt/GSK-3β signaling pathway could be an important therapeutic target for neuroprotection against cerebral ischemia injury (Miyawaki et al., 2009; Lu et al., 2011; Dong et al., 2021). The PI3K/Akt/GSK-3β signaling pathway participates in antiapoptotic cell death *via* acting on Bcl-2 and inhibiting caspase-3 (Hanada et al., 2004). In our study, treatment of MC-ELNs increased the ratio of Bcl-2/Bax in the cortex, inhibited cleaved caspase-3, and reduced apoptosis by TUNEL staining in the striatum and hippocampus of the rats after being exposed to cerebral I/R injury (Figure 6). Furthermore, MC-ELNs significantly upregulated the expression of the phosphorylated AKT (Ser473) and GSK-3β (Ser9) in the cultured HT22 cells under OGD/R exposure, subsequently inhibiting apoptosis by flow cytometry (Figure 7). Therefore, we conclude that MC-ELNs could protect against cerebral I/R injury by regulating the AKT/GSK-3β signaling pathway.

In summary, to our knowledge, this study is the first to explore plant-derived exosome-like nanoparticles used as a therapeutic agent for ischemic stroke. MC-ELNs, exosome-like nanoparticles from *Momordica charantia,* have the capacity of crossing the BBB, reducing infarct size, and improving neurological deficits in the cerebral ischemia-reperfusion injury. The underlying mechanisms could be attributed to the inhibition of MMP-9 and upregulation of the AKT/GSK-3β signaling pathway. Furthermore, miR5266 could be one of the active components derived from MC-ELNs to inhibit MMP-9, which remains to be further studied. At present, we still know little about the other bioactive components in MC-ELNs in addition to small RNAs. On the other hand, it remains elusive whether other downstream molecules of miR5266 also contribute to BBB protection. Also, to obtain a substantial conclusion, we are still planning to confirm the effects of miR5266 in the stroke model.

## Data Availability

The datasets presented in this study can be found in online repositories. The names of the repository/repositories and accession number(s) can be found at: https://www.ncbi.nlm.nih.gov/sra/PRJNA826311.

## Abbreviations

t-PA, tissue plasminogen activator; ELNs, exosome-like nanoparticles; MC, *Momordica charantia*; MCAO, middle cerebral artery occlusion; BBB, blood–brain barrier; MMP-9, matrix metalloproteinase 9; ZO-1, zona occluden 1 protein; Bcl-2, B-cell lymphoma-2; AKT, serine/threonine kinase; GSK3β, glycogen synthase kinase 3β.
