# Supplementary material for: Momordica charantia Exosome-Like Nanoparticles Exert Neuroprotective Effects Against Ischemic Brain Injury via Inhibiting Matrix Metalloproteinase 9 and Activating the AKT/GSK3β Signaling Pathway
Source: Front Pharmacol. 2022 Jun 24;13:908830. doi: 10.3389/fphar.2022.908830 (PMC9263912; doi:10.3389/fphar.2022.908830)
Supplement: Supplementary file 1 [file DataSheet1.doc]

**Supplementary information**


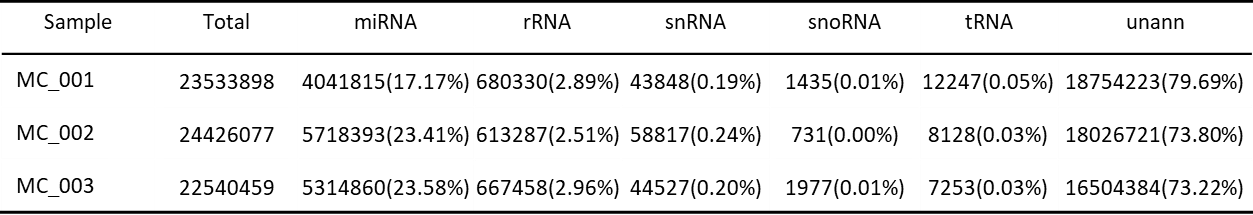


Table S1. Total tag classification annotation

Table S2. Unique tag classification annotation


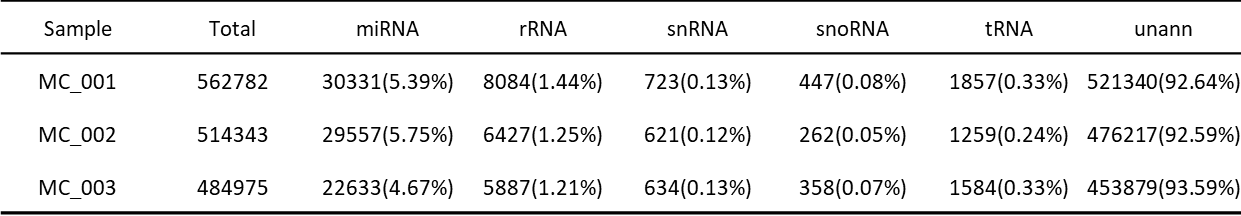


Table S3. The miRNA expression of all samples

| miRNA_name | Sequence | Length | Total Count | MC_001  count | MC_002  count | MC_003  count |
| --- | --- | --- | --- | --- | --- | --- |
| miR10210-5p | GTTGTCTATGTTCGTCAGTA | 20 | 755 | 0 | 0 | 755 |
| miR10218-3p | ACTGGGAAGAGACCTGACT | 19 | 135 | 135 | 0 | 0 |
| miR10218-5p | TCGGGCGCCCCCCAGATTG | 19 | 207 | 44 | 163 | 0 |
| miR11066-5p | TAAGTGCTTCCATGTTTG | 18 | 4982 | 3152 | 0 | 1830 |
| miR11071-3p | TCGATTAAGACAGTAGGAC | 19 | 209788 | 0 | 209788 | 0 |
| miR11082a-3p | CAAGGAGTTGTTGGACTG | 18 | 796 | 0 | 0 | 796 |
| miR1110 | GCAGGACAGTGGTCATGGA | 19 | 10310 | 2207 | 1428 | 6675 |
| miR11104-3p | GGGTTGGGTCGGGATTAA | 18 | 858 | 0 | 858 | 0 |
| miR11108a-5p | TTGTCTAATGACCTCAGTA | 19 | 58 | 0 | 0 | 58 |
| miR11155b-5p | CAAGGAGTTGGATTAGTCCCTC | 22 | 201 | 0 | 0 | 201 |
| miR11323 | TTGCATTGCACACTCAGTG | 19 | 64 | 0 | 0 | 64 |
| miR11420c | CCGGAGGACCCGGTCGGTCC | 20 | 620 | 218 | 171 | 231 |
| miR1147.1 | GAGTGCTCAGCGCGCTCCCGGCG | 23 | 1432 | 310 | 1122 | 0 |
| miR11481a | GTGTTCGGACGGTCGGTAGC | 20 | 73 | 0 | 0 | 73 |
| miR1150.3 | TCGCGGCGACGTGGGGCCGG | 20 | 9870 | 0 | 0 | 9870 |
| miR11539a | GGCAGCAGGCGCGATTAAGAC | 21 | 1089 | 485 | 604 | 0 |
| miR12175 | TTGGATCAGTTCTTCAACG | 19 | 27 | 0 | 0 | 27 |
| miR1520n | TCAACTCAGAACTGGTACGGACA | 23 | 390 | 0 | 0 | 390 |
| miR164c-3p | CCCTGTCTACTATCCAAC | 18 | 59773 | 0 | 59773 | 0 |
| miR166a-3p | TCGGACCAGGCTTCATTCCCC | 21 | 32 | 0 | 32 | 0 |
| miR166c-5p | GGGGAATGTTGTCTGGTGCGAGA | 23 | 45 | 0 | 0 | 45 |
| miR166h-3p | TCGGACCAGGCTTCATTCCC | 20 | 48 | 0 | 0 | 48 |
| miR166l-5p | GCGATTTGTCTGGTTAAAGG | 20 | 14629 | 8843 | 5786 | 0 |
| miR167d-3p | TCGGATCATGTGTGTTCATCC | 21 | 135 | 0 | 135 | 0 |
| miR169d-5p | AAGCCAAGGATGACTTGCCGG | 21 | 26 | 26 | 0 | 0 |
| miR171h-5p | GTTGGGATCGTCAATCAGTA | 20 | 135 | 0 | 0 | 135 |
| miR1846a-5p | AGTGGGAGCCGGGGCGCC | 18 | 2933 | 0 | 2933 | 0 |
| miR1848 | GCTCCCGGCGCGCGCGGA | 18 | 1259 | 0 | 1259 | 0 |
| miR2096-3p | CCGCGGGGAAAGCGGCGGGTA | 21 | 478 | 0 | 0 | 478 |
| miR2102-5p | GGCCTGCCGCCGCGCCAC | 18 | 263 | 0 | 0 | 263 |
| miR2118-5p | GGTCGATGGAACAATGTAGGCAAGG | 25 | 15534 | 15534 | 0 | 0 |
| miR2594 | GCATGGCCAAGGTCCAGAC | 19 | 112 | 0 | 0 | 112 |
| miR2613 | CGGTCGGTCGGTGGTCATGGA | 21 | 1052 | 0 | 1052 | 0 |
| miR2864.2 | TTGTTTTGCCTTGTCAGTA | 19 | 274 | 0 | 0 | 274 |
| miR2867-3p | CCAGGACGGTGGTCATGGA | 19 | 138473 | 40932 | 43893 | 53648 |
| miR3435-3p | AGTTGACAGTATTATTCAGTA | 21 | 191 | 0 | 0 | 191 |
| miR3710d | GTGGGAGCCGAGGGGCCTC | 19 | 441 | 0 | 441 | 0 |
| miR3932b-5p | TTGGACTGCTCGAGCTGCT | 19 | 287467 | 0 | 0 | 287467 |
| miR398b-3p | TGTGTTCTCAGGTAACCCCTG | 21 | 42 | 0 | 0 | 42 |
| miR398b-5p | GGGGCAGGACTGGGAAGATTG | 21 | 72513 | 37852 | 33180 | 1481 |
| miR399h | GGGCACCACCGCTTGGCA | 18 | 759 | 701 | 0 | 58 |
| miR408 | GCGGGGCGAAGCCAGAGCAG | 20 | 1333817 | 362166 | 208506 | 763145 |
| miR472-5p | AGGGAAGTCGGCAAAATC | 18 | 735 | 735 | 0 | 0 |
| miR482b-3p | CCTGCCCACCCCCCCATTCG | 20 | 3463 | 0 | 746 | 2717 |
| miR5017-5p | ATTGTTACTATTGGATTG | 18 | 10 | 10 | 0 | 0 |
| miR5044 | GTAGTGGAGCCTGGAAGCCA | 20 | 156 | 0 | 0 | 156 |
| miR5054 | GACCCCAGGTCGGCGGCCA | 19 | 1933 | 1933 | 0 | 0 |
| miR5059 | GCCTTGGCCGCACCACCA | 18 | 8674 | 2602 | 2341 | 3731 |
| miR5061 | TTGTACTGTCCTCTCAGTA | 19 | 507 | 0 | 0 | 507 |
| miR5074 | ACGGCCACCTTCGGGATCG | 19 | 19 | 19 | 0 | 0 |
| miR5266 | CGGGGGACGGACTGGGCG | 18 | 451853 | 115859 | 74917 | 261077 |
| miR536 | CCGCGGCCAGGTTGCACCACC | 21 | 503 | 0 | 0 | 503 |
| miR536a | CCGCGCCAAGCGCGCGACC | 19 | 1103 | 0 | 1103 | 0 |
| miR5386 | CGTCCCTGCCCGCGCGCT | 18 | 2549 | 0 | 0 | 2549 |
| miR5565e | TTGTTTGTATTTGTCAGTA | 19 | 335 | 0 | 0 | 335 |
| miR5788 | TTGATGTGCATACTCAGTG | 19 | 65 | 0 | 0 | 65 |
| miR5800 | CCCGGCATCGGAACGGCCTGCC | 22 | 85 | 0 | 42 | 43 |
| miR5813 | AGCAGGACGGTGGTCATGGA | 20 | 4015235 | 995811 | 1017454 | 2001970 |
| miR6135c | GGGTGTTGGTCAATTAAGAC | 20 | 8328554 | 2415375 | 4011623 | 1901556 |
| miR6151f | GGAGTTGTGGCATTGGATTG | 20 | 203 | 0 | 0 | 203 |
| miR6168 | CCTTGGCCGCGGTGGACTGCTCG | 23 | 10867 | 3951 | 3546 | 3370 |
| miR6221-3p | CCGCGGCCAGATTAAGACAGC | 21 | 5001 | 0 | 5001 | 0 |
| miR6222-5p | CCTGGTGGGATAGCCAAGG | 19 | 81 | 0 | 0 | 81 |
| miR6250 | GGGGATAGACGAGGCGCTGTCAACG | 25 | 457 | 0 | 0 | 457 |
| miR6300 | GTCGTTGTAGTATAGTGGTA | 20 | 78 | 0 | 78 | 0 |
| miR6478 | CCGACCTTAGCTCAGTTGGTAG | 22 | 6811 | 3128 | 2713 | 970 |
| miR7532a | GAACAGCCTCTGGTCGATGGA | 21 | 17941 | 13347 | 3733 | 861 |
| miR7533d-3p | GACTGAAGGAGAGGATCC | 18 | 42 | 0 | 0 | 42 |
| miR7539 | GCGAGAGAGAGAGAGAGAGAG | 21 | 405 | 103 | 133 | 169 |
| miR7584e | AGGAGTTGATTGTAGTTTTCAGTA | 24 | 797 | 0 | 0 | 797 |
| miR7768a-5p | CCCGATCGTGGCGGCCCCC | 19 | 304 | 0 | 130 | 174 |
| miR8175 | TCCCCGGCAACGGCGCCA | 18 | 660 | 0 | 370 | 290 |
| miR8176 | GGACGGTGGTCACAAGGGA | 19 | 4886 | 1863 | 0 | 3023 |
| miR858 | TCTCGTTGTCTGTTCGACCGATTG | 24 | 41 | 41 | 0 | 0 |
| miR902k-5p | AGTTGCAGATTCTTGATTG | 19 | 5 | 5 | 0 | 0 |
| miR914 | CGGGCGCCCGAATCCGGGC | 19 | 1240 | 956 | 284 | 0 |
| miR948 | TGTGGTCTTTGGGTTCCG | 18 | 14926 | 0 | 14926 | 0 |
| miR9484 | TAGGCAAGGGAAGTCGGC | 18 | 25859 | 15070 | 10789 | 0 |
| miR9499 | CCGCCCGTCGACGGCGGCCAGCTC | 24 | 1428 | 238 | 1190 | 0 |
| miR950h | GCACCACCGGGCCCCGATG | 19 | 1710 | 310 | 167 | 1233 |
| miR9725 | TTGATTTTTTTGATCAGTA | 19 | 328 | 0 | 0 | 328 |

**Supplementary file S1. mNSS score**

| **Motor tests** |  |
| --- | --- |
| ***Raising rat by tail*** | 3 |
| Flexion of forelimb | 1 |
| Flexion of hindlimb | 1 |
| Head moved 10° to vertical axis within 30 s | 1 |
| ***Placing rat on floor (normal0; maximum3)*** | 3 |
| Normal walk | 0 |
| Inability to walk straight | 1 |
| Circling toward the paretic side | 2 |
| Falls down to the paretic side | 3 |
| **Sensory tests** | 2 |
| Placing test (visual and tactile test) | 1 |
| Proprioceptive test (deep sensation, pushing paw against table edge to stimulate limb muscles) | 1 |
| **Beam balance tests (normal0; maximum6)** | 6 |
| Balances with steady posture 0 | 0 |
| Grasps side of the beam | 1 |
| Hugs beam and 1 limb falls down from the beam | 2 |
| Hugs beam and 2 limbs fall down from the beam, or spins on beam (60 s) | 3 |
| Attempts to balance on beam but falls off (40 s) | 4 |
| Attempts to balance on beam but falls off (20 s) | 5 |
| Falls off; no attempt to balance or hang on to beam (20 s) | 6 |
| **Reflex absence and abnormal movements** | 4 |
| Pinna reflex (head shake when auditory meatus is touched) | 1 |
| Corneal reflex (eye blink when cornea is lightly touched with cotton) | 1 |
| Startle reflex (motor response to a brief noise from snapping a clipboard paper) | 1 |
| Seizures, myoclonus, myodystony | 1 |
| **Maximum points** | 18 |

**Supplementary file S2**

Rat mmp-9 sequence

NM_013599-3’UTR

AACCCCATCCGAGGGAAAGGTGCTAGCTGGCCAGGTACAGACTGGTGATCTCTTCTAGAGACTGGGAAGGAGTGGAGGCAGGCAGGGCTCTCTCTGCCCACCGTCCTTTCTTGTTGGACTGTTTCTAATAAACACGGATCCCCAACCTTTTCCAGCTACTTTAGTCAATCAGCTTATCTGTAGTTGCAGATGCATCCGAGCAAGAAGACGACAACTTTGTAGGGTGGATTCTGACCTTTTATTTTTGTGTGGCGTCTGAGAATTGAATCAGCTGGCTTTTGTGACAGGCACTTCACCGGCTAAACCACCTCTCCCGACTCCAGCCCTTTTATTTATTATGTATGAGGTTATGTTCACATGCATGTATTTAACCCACAGAATGCTTACTGTGTGTCGGGCGCGGCTCCAACCGCTGCCTGGGCGTTAGGGACAGAAATGTTGGTTCTTCCTTCAAGGATTGCTCAGAGATTCTCCGTGTCCTGTAAATCTGCTGAAACCAGACCCCAGACTCCTCTCTCTCCCGAGAGTCCAACTCACTCACTGTGGTTGCTGGCAGCTGCAGCATGCGTATACAGCATGTGTGCTAGAGAGGTAGAGGGGGTCTGTGCGTTATGGTTCAGGTCAACTCACTCACTGTGGTTGCTGGCAGCTGCAGCATGCGTATACAGCATGTGTGCTAGAGAGGTAGAGGGGGTCTGTGCGTTATGGTTCAGGTCAGACTGTGTCCTCCAGGTGAGATGACCCCTCAGCTGGAACTGATCCAGGAAGGATAACCAAGTGTCTTCCTGGCAGTCTTTTTTAAATAAATGAATAAATGAATATTTACTTAT

Red: putative miRNA5266 binding sites in MMP-9 3’UTR

NM_013599-3’UTR-mut

AACCCCATCCGAGGGAAAGGTGCTAGCTGGCCAGGTACAGACTGGTGATCTCTTCTAGAGACTGGGAAGGAGTGGAGGCAGGCAGGGCTCTCTCTGCCCAAATGAAGGGCTTGTTGGACTGTTTCTAATAAACACGGATCCCCAACCTTTTCCAGCTACTTTAGTCAATCAGCTTATCTGTAGTTGCAGATGCATCCGAGCAAGAAGACGACAACTTTGTAGGGTGGATTCTGACCTTTTATTTTTGTGTGGCGTCTGAGAATTGAATCAGCTGGCTTTTGTGACAGGCACTTCACCGGCTAAACCACCGAGAAATACTCCAGCCCTTTTATTTATTATGTATGAGGTTATGTTCACATGCATGTATTTAACCCACAGAATGCTTACTGTGTGTCGGGCGCGGCTCCAACCGCTGCCTGGGCGTTAGGGACAGAAATGTTGGTTCTTCCTTCAAGGATTGCTCAGAGATTCTCCGTGTCCTGTAAATCTGCTGAAACCAGACCCCAGACTCCTCTCGAGAAATAGAGTCCAACTCACTCACTGTGGTTGCTGGCAGCTGCAGCATGCGTATACAGCATGTGTGCTAGAGAGGTAGAGGGGGTCTGTGCGTTATGGTTCAGGTCAACTCACTCACTGTGGTTGCTGGCAGCTGCAGCATGCGTATACAGCATGTGTGCTAGAGAGGTAGAGGGGGTCTGTGCGTTATGGTTCAGGTCAGACTGTTGAAGAAAGGTGAGATGACCCCTCAGCTGGAACTGATCCAGGAAGGATAACCAAGTGTCTTCCTGGCAGTCTTTTTTAAATAAATGAATAAATGAATATTTACTTAT

Green: mutant miRNA5266 binding sites in MMP-9 3’UTR
